# Differential Effects of Methyl-4-Phenylpyridinium Ion, Rotenone, and Paraquat on Differentiated SH-SY5Y Cells

**DOI:** 10.1155/2013/347312

**Published:** 2013-03-20

**Authors:** João Barbosa Martins, Maria de Lourdes Bastos, Félix Carvalho, João Paulo Capela

**Affiliations:** ^1^REQUIMTE (Rede de Química e Tecnologia), Laboratório de Toxicologia, Departamento de Ciências Biológicas, Faculdade de Farmácia, Universidade do Porto, Rua de Jorge Viterbo Ferreira 228, 4050-313 Porto, Portugal; ^2^Faculty of Health Sciences, University Fernando Pessoa, Rua Carlos da Maia 296, 4200-150 Porto, Portugal

## Abstract

Paraquat (PQ), a cationic nonselective bipyridyl herbicide, has been used as neurotoxicant to modulate Parkinson's disease in laboratory settings. Other compounds like rotenone (ROT), a pesticide, and 1-methyl-4-phenylpyridinium ion (MPP^+^) have been widely used as neurotoxicants. We compared the toxicity of these three neurotoxicants using differentiated dopaminergic SH-SY5Y human cells, aiming to elucidate their differential effects. PQ-induced neurotoxicity was shown to be concentration and time dependent, being mitochondrial dysfunction followed by neuronal death. On the other hand, cells exposure to MPP^+^ induced mitochondrial dysfunction, but not cellular lyses. Meanwhile, ROT promoted both mitochondrial dysfunction and neuronal death, revealing a biphasic pattern. To further elucidate PQ neurotoxic mechanism, several protective agents were used. SH-SY5Y cells pretreatment with tiron (TIR) and 2-hydroxybenzoic acid sodium salt (NaSAL), both antioxidants, and *N*_**ω**_-nitro-L-arginine methyl ester hydrochloride (L-NAME), a nitric oxide synthase inhibitor, partially protected against PQ-induced cell injury. Additionally, 1-(2-[bis(4-fluorophenyl)methoxy]ethyl)-4-(3-phenyl-propyl)piperazine (GBR 12909), a dopamine transporter inhibitor, and cycloheximide (CHX), a protein synthesis inhibitor, also partially protected against PQ-induced cell injury. In conclusion, we demonstrated that PQ, MPP^+^, and ROT exerted differential toxic effects on dopaminergic cells. PQ neurotoxicity occurred through exacerbated oxidative stress, with involvement of uptake through the dopamine transporter and protein synthesis.

## 1. Introduction

Parkinson's disease (PD) is considered the second most common neurodegenerative disorder worldwide, affecting 0.5 to 1% of the population aged between 65 and 69 years and 1 to 3% of the population over 80 years [1]. PD develops from a loss of nigrostriatal neuromelanin-containing dopaminergic neurons, whose cell bodies lay in the substantia nigra pars compacta (SNpc) [2]. This nigrostriatal pathway is essential for a normal motor function and movement control. PD is thought to have a multifactorial etiology, frequently including genetic and environmental factors [2, 3]. Several neurotoxic chemicals to dopaminergic neurons leading to PD-like symptoms have been used to study this disease. The synthetic compounds 1-methyl-4-phenyl-1,2,3,6-tetrahydropyridine (MPTP) and 1-methyl-4-phenyl-4-propionoxy-piperidine (MPPP) were the first to be associated with PD symptoms, as described by Langston and Ballard [4]. MPTP enters the blood-brain barrier and is metabolized in glial cells by monoamine oxidases to 1-methyl-4-phenyl-2,3-dihydropyridium (MPDP^+^), which is subsequently oxidized to MPP^+^ [5]. Next, MPP^+^ enters the dopaminergic cell via dopamine transporter (DAT) [5], accumulates inside the mitochondria, and interferes with complex I of mitochondrial transport chain, inhibiting its activity [6]. This reduces ATP cellular stores, promoting reactive oxygen species (ROS) formation and consequentially leading to neuronal death [5]. Another substance known to promote Parkinsonism is the organic pesticide rotenone (ROT), which is used worldwide as an insecticide and to eliminate nuisance fish populations, in lakes and reservoirs [3]. It is used to study PD given its ability to cross biological membranes, not depending on transporters, inhibiting mitochondrial complex I [7] which increases the rate of mitochondrial ROS release, leading to cell apoptosis [3, 8]. Despite their use as PD models, neither ROT nor MPP^+^ were correlated with the sporadic occurrence of PD [8]. These two neurotoxicants are currently used to study PD by means of *in vivo* and* in vitro *approaches; however, none of these substances reproduces completely all the clinical features observed in PD [8, 9]. 

The lack of adequate cellular and/or animal models of PD has prompted the screening of many putative neurotoxic compounds. Paraquat (PQ) has received recently a wide attention as a possible inducer of PD. In fact, occupational exposure, especially in farming, has been associated with Parkinsonism [10] and also, it was demonstrated that patients who died from PQ poisoning had severe brain damage [11]. This was corroborated by studies, in animals, where besides the affection of the lung which is the main target organ for PQ toxicity, it was also demonstrated to be toxic to dopaminergic neurons [12, 13]. Several mechanisms have been postulated for PQ-induced neurotoxicity, including increase in ROS formation, excitotoxicity, and mitochondrial complex I inhibition [14–16]. Nonetheless, the mechanisms by which PQ promotes neurotoxicity still remain to be fully elucidated. 

The present work aimed to study the differences in terms of dopaminergic neurotoxic profile among PQ, ROT, and MPP^+^ in a cell culture model with a dopaminergic phenotype. Also, we intended to provide further insights into the mechanism of PQ-induced neurotoxicity. For that purpose, human dopaminergic SH-SY5Y-differentiated cells were used.

## 2. Materials and Methods

### 2.1. Materials

The reagents for cell culture were obtained from Gibco (Invitrogen, Paisley, UK): Dulbecco's Modified Eagle Medium (D-MEM), nonessential amino acids (NEAA), phosphate-buffered saline (PBS), trypsin/EDTA, and penicillin/streptomycin. 48 Multiwell plates and 35 mm plates were obtained from Corning Costar (Corning, NY, USA). 25 cm^3^ flasks were obtained from TPP (Trasadingen, Switzerland); Fuchs-Rosenthal Counting Chamber was obtained from Carl-Rhode (Germany). Other reagents, namely, trypan blue solution (0.4%), dimethyl sulfoxide (DMSO), retinoic acid (RA), 12-*O*-tetradecanoyl-phorbol-13-acetate (TPA), 3-[4,5-dimethylthiazol]-2,5-diphenyltetrazolium (MTT), sodium dodecyl sulfate (SDS), enzyme-standard for kinetic lactate dehydrogenase (LDH)-assay, *β*-nicotinamide adenine dinucleotide reduced form (*β*-NADH), 1,1′-dimethyl-4,4′-bipyridinium dichloride (Paraquat or PQ), rotenone (ROT), 1-methyl-4-phenyl-pyridinium ion (MPP^+^), *N*-acetylcysteine (NAC), tiron (TIR), 1-(2-[bis(4-fluorophenyl)methoxy]ethyl)-4-(3-phenyl-propyl)piperazine (GBR 12909), cycloheximide (CHX), 2-hydroxybenzoic acid sodium salt (NaSAL), and *N *_*ω*_-nitro-L-arginine methyl ester hydrochloride (L-NAME), were obtained from Sigma-Aldrich (St Louis, MO, USA).

### 2.2. Cell Line and Culture Conditions

Human neuroblastoma SH-SY5Y cells (ATCC, Manassas, VA, USA) were used. Through differentiation (see protocol below), SH-SY5Y cells are able to express dopaminergic markers, as well as tyrosine hydroxylase, DAT, and higher ability to accumulate dopamine and exhibit extended neurites [17, 18]. In this way, the cells are no longer immature, mitosis rate is reduced, and also differentiation agents can mimic factors secreted by astrocytes in a the natural brain environment. In other words, differentiation can provide a cellular model more similar to dopaminergic neurons [17, 18]. The cells were grown in D-MEM containing 4.5 mg/L D-glucose, 2 mM L-glutamine, 110 mg/L sodium pyruvate, phenol red supplemented with 10% fetal bovine serum, 100 *μ*g/mL penicillin, 100 *μ*g/mL streptomycin, and 1% NEAA, under an atmosphere of 5% CO_2_/95% air at 37°C. 

### 2.3. Experimental Protocol

Stock cultures of SH-SY5Y cells (passages 20 to 45) were maintained in 25 cm^3^ flasks and grown until confluence (70–80% confluence). Cells were washed with PBS, trypsinized (trypsin/EDTA 0.05/0.02% w/v), counted by trypan blue exclusion, using a Fuchs-Rosenthal counting chamber, and subcultured at the density of 25 000 cells/cm^2^ in 48-well culture plates. SH-SY5Y cells were differentiated, in order to enhance the dopaminergic phenotype, by the addition of 10 *μ*M RA to the medium for 3 days, after which the medium was added with 70 nM TPA and kept for another 3 days, in accordance with previous works [17, 18]. Finally, the medium was removed and replaced with 250 *μ*L fresh medium alone or with the drugs: PQ (100, 500, and 1000 *μ*M), MPP^+^(100, 500, and 1000 *μ*M) and ROT (1, 10, and 100 *μ*M). Concentrations of the neurotoxicants were selected in accordance with previous studies [19–24]. The cells were exposed to the drugs for different time periods (24, 48, and 72 h). ROT was initially prepared in DMSO and then diluted in medium in order to obtain a final concentration of 0.1% of DMSO in the culture medium, which was not toxic to cells. To avoid variations among cell cultures that could interfere in the toxicity outcome, results for the concentration-toxicity curves of the 3 toxins were obtained from several cell cultures resulting from different passages and were seeded in different days. Cell culture procedures and validation of the differentiation protocol of SH-SY5Y cells were previously published by our group [18]. After selecting the concentration and time of exposure through screening experiments, the antioxidants NAC (1 mM) [25], and TIR (100 *μ*M and 1 mM) [26], the specific DAT inhibitor GBR 12909 (1 *μ*M) [23], the protein synthesis inhibitor CHX (1.8 nM) [27], the radical scavenger NaSAL (100 *μ*M and 1 mM) [28], and the nonselective nitric oxide synthase (NOS) inhibitor L-NAME (1 *μ*M) [29] were added 30 minutes before exposure of cells to PQ. These concentrations of the pharmacological antagonists were selected according to the drug selectivity to the target and also taking into account previous cell culture studies mentioned above. 

### 2.4. Life-Death Assays

 Cell cultures were assessed morphologically by phase contrast microscopy at 3 different time points (24, 48, and 72 h). Cell damage was assessed quantitatively by the measurement of LDH release into the medium (as a measure of cell membrane integrity) using a kinetic measurement assay. Also, mitochondrial dysfunction was assessed by the measurement of MTT salt metabolism assay. 

#### 2.4.1. Lactate Dehydrogenase Kinetic Assay

The quantification of LDH activity was made by a colorimetric method, which is based on the reversible reduction of pyruvate to lactate in the presence of *β*-NADH, as described by Capela et al., 2006. After exposure to drugs, two aliquots (50 *μ*L each) of the medium were removed, to which a previously prepared 0.15 mg/mL *β*-NADH solution was added, at room temperature, in a 96-well microplate. Finally, pyruvate 22.7 mM was added to start the reaction. NADH oxidation to NAD^+^ was measured at 340 nm, using a colorimetric 96-well plate reader (BioTek Instruments, VT, USA). The delta increase in LDH release into the medium was calculated by subtraction of the respective controls, which were untreated cells cultured alongside the treated cells. LDH values are expressed in units per liter (U/L), calculated based on a LDH standard solution activity of 500 U/L [29].

#### 2.4.2. MTT Assay

 This colorimetric assay relies on the ability of viable, but not dead cells to convert a soluble yellow tetrazolium dye, MTT, into an insoluble blue formazan product that can be measured at 550 nm. Given the removal of 100 *μ*L of culture medium used for the LDH assay, each well was added 50 *μ*L fresh medium to obtain 200 *μ*L of cell medium per well, to which the MTT solution was added (final concentration of MTT 500 *μ*g/mL). Subsequently, cells were incubated at 36.5°C for 3 hours. The reaction was stopped by adding an equal volume of 10% SDS in 0.01 M hydrochloric acid solution followed by an overnight incubation at 36.5°C. Finally, formazan was detected at 550 nm using a colorimetric 96-well plate reader, as previously described [29]. The viability of untreated control cells was set to 100%, and the effects resulting from toxicant exposure were expressed as the percentage of the respective controls.

### 2.5. Statistical Analysis

 Results are presented as mean ± S.E.M., from at least 3 different independent experiments. The means for concentration/time graphics were compared using the two-way ANOVA, followed by the Bonferroni post hoc test, once a significant *P* had been obtained. The means for the tested neuroprotectors were compared using the one-way ANOVA, followed by Student-Newman-Keuls post hoc test, once a significant *P* had been obtained. Details of the statistical analyses are described in each figure legend. Significance was accepted when *P* was less than 0.05.

## 3. Results

### 3.1. Paraquat Was Toxic to SH-SY5Y Cells in a Concentration- and Time-Dependent Manner

**Figure 1:**
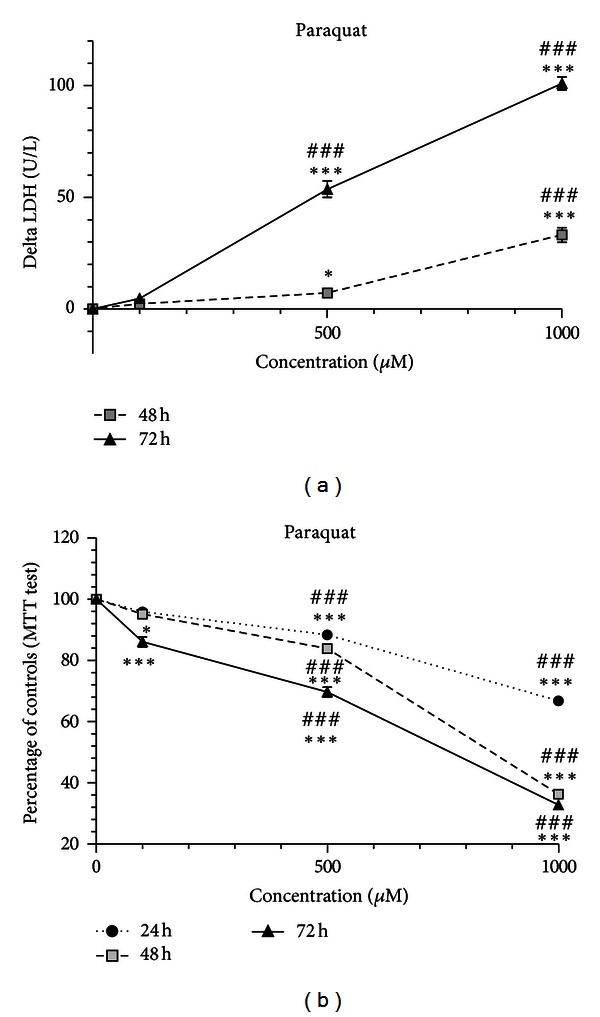
PQ toxicity occurred in a concentration- and time-dependent manner in differentiated SH-SY5Y cells. Cells were incubated with 100, 500 and 1000 *μ*M of PQ for 24, 48 and 72 h. (a) Increase in LDH release into the medium in differentiated SH-SY5Y cells. The delta increase in LDH release in units per liter (U/L) into the medium was calculated by subtraction of the respective controls (results were pooled from 3 different experiments, each experiment having 6 different culture wells per condition). (b) Mitochondrial dysfunction in differentiated SH-SY5Y cells evaluated by the MTT test. The viability of untreated control cells was set to 100%, and all treatments were expressed as the percentage of the respective controls (results were pooled from 3 different experiments, each experiment having 6 different culture wells per condition). The means were compared using the two-way ANOVA test, followed by the Bonferroni post hoc test (*P < 0.05, ***P < 0.001 concentration versus control; ^###^ *P* < 0.001 concentration versus concentration).

The exposure of differentiated SH-SY5Y cells to PQ (100 *μ*M, 500 *μ*M and 1000 *μ*M) for 24 h, did not result in elevation of LDH release at any concentration tested (data not shown). However, at 48 h there was a significant LDH release with 500 *μ*M, which was even more marked with 1000 *μ*M exposure. As expected, LDH release into the medium was more pronounced at 72 h, for 500 and 1000 *μ*M. Thus, the loss of cellular viability occurred in a concentration- and time-dependent manner (Figure 1(a)). Mitochondrial dysfunction was assayed by the MTT assay. In contrast to cellular death evaluated by the LDH release, mitochondrial dysfunction was already visible at the 24 h time-point for the 500 *μ*M and 1000 *μ*M concentrations. For the 100 *μ*M concentration, PQ decreased significantly MTT reduction at 48 h but especially at 72 h. Therefore, it could be clearly observed that mitochondrial dysfunction also occurred in a concentration- and time-dependent manner (Figure 1(b)). 

### 3.2. MPP^+^-Induced Mitochondrial Dysfunction in SH-SY5Y Cells

**Figure 2:**
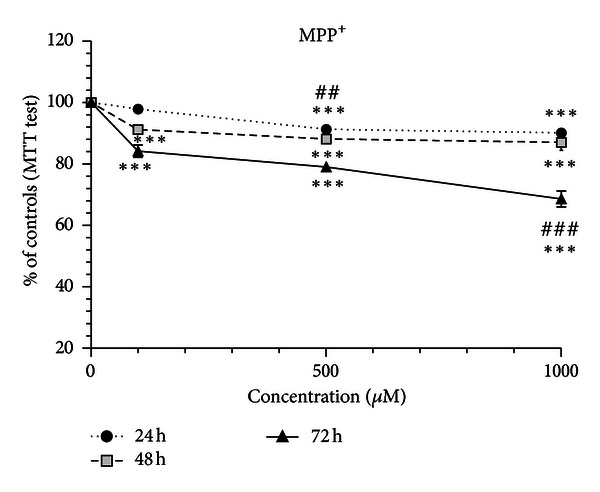
MPP^+^-induced mitochondrial dysfunction in differentiated SH-SY5Y. The cells were incubated with 100, 500, and 1000 *μ*M of MPP^+^, and the MTT test was performed at 24, 48, and 72 h time-points. Mitochondrial dysfunction in differentiated SH-SY5Y cells evaluated by the MTT test. The viability of untreated control cells was set to 100%, and all treatments were expressed as the percentage of the respective controls (results were pooled from 3 different experiments, each experiment having 6 different culture wells per condition). The means were compared using the two-way ANOVA, followed by the Bonferroni post hoc test (***P < 0.001 concentration versus control; ^##^ *P* < 0.01, ^###^ *P* < 0.001 concentration versus concentration).

Following exposure of SH-SY5Y cells to MPP^+^ (100 *μ*M, 500 *μ*M, and 1000 *μ*M), there was no increase in cell injury at any time-point or concentration tested, as assessed by the LDH release into the medium (data not shown). On the other hand, MPP^+^ proved to induce mitochondrial dysfunction (Figure 2). In fact, there was significant mitochondrial dysfunction already after 24 h of exposure to 500 *μ*M, and 1000 *μ*M concentrations. There was no apparent increase in the toxicity by extending the exposure period to 48 h. However, at 72 h MTT metabolism decreased significantly for all concentrations tested, especially for 1000 *μ*M. Only at this point the toxicity of 1000 *μ*M was significantly higher than 500 *μ*M (Figure 2). Thus, only longer periods of exposure resulted in a concentration-dependent effect.

### 3.3. Rotenone Induced a Biphasic Pattern of Neurotoxicity in SH-SY5Y Cells

**Figure 3:**
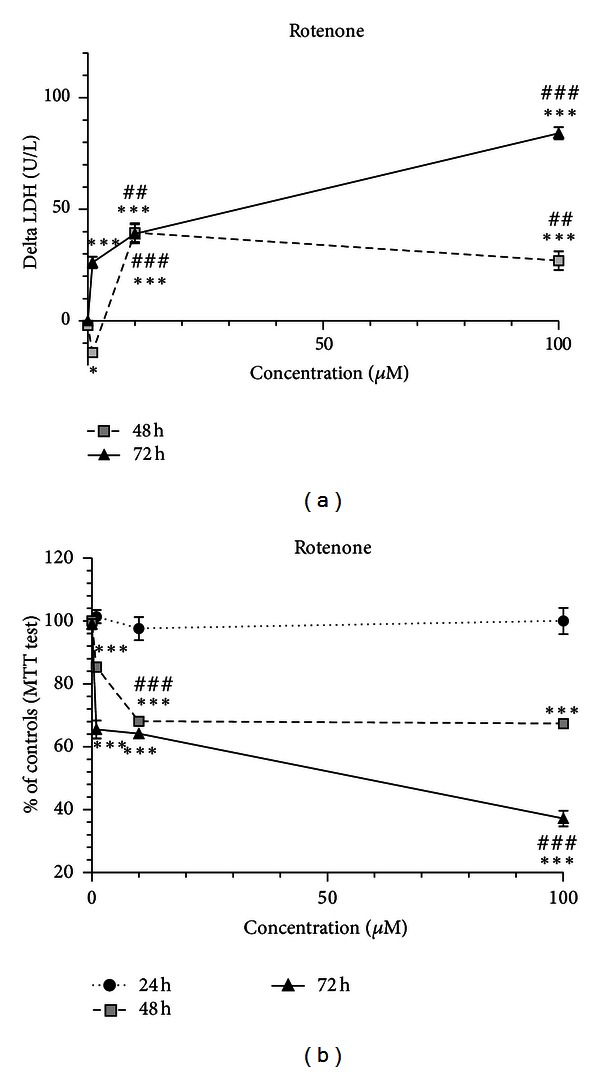
ROT-induced neurotoxicity showed a biphasic pattern with mitochondrial dysfunction and cell death in differentiated SH-SY5Y cells. The cells were incubated with 1, 10, and 100 *μ*M of ROT, and then MTT and LDH tests were performed at 24, 48, and 72 h time points. (a) Increase in LDH release in units per liter (U/L) into the medium in differentiated SH-SY5Y cells. The delta increase in LDH release into the medium was calculated by subtraction of the respective controls (results were pooled from 3 different experiments, each experiment having 6 different culture wells per condition). (b) Mitochondrial dysfunction in differentiated SH-SY5Y cells evaluated by the MTT test. The viability of untreated control cells was set to 100%, and all treatments were expressed as the percentage of the respective controls (results were pooled from 3 different experiments, each experiment having 6 different culture wells per condition). The means were compared using the two-way ANOVA, followed by the Bonferroni post hoc test (***P < 0.001 concentration versus control; ^##^ *P* < 0.01, ^###^ *P* < 0.001 concentration versus concentration).

Exposure of SH-SY5Y cells to ROT (1 *μ*M, 10 *μ*M, and 100 *μ*M) did not result in LDH release into the medium, at the 24 h time-point (data not shown). Interestingly, ROT exhibited a biphasic pattern of cellular lyses, at the 48 h time-point (Figure 3(a)). At this time-point, 1 *μ*M ROT led to a significant reduction of LDH release into the medium, to levels below those of controls. Meanwhile, the 10 *μ*M concentration elicited an increase in LDH release, which was significantly higher than that observed for 100 *μ*M. Thus, cell injury occurred in a concentration-independent manner. After 72 h exposure there was a concentration-dependent toxicity. Curiously, values for LDH release at 48 h and 72 h for the 10 *μ*M concentration were very similar (Figure 3(a)).

Exposure for 24 h to 10 *μ*M, and 100 *μ*M ROT did not induce mitochondrial dysfunction (Figure 3(b)). After 48 h exposure there was an increase of mitochondrial dysfunction from 1 *μ*M to 10 *μ*M, in which values were similar to those of the 100 *μ*M concentration. However, after 72 h exposure there was a concentration-dependent toxicity, with the highest levels of mitochondrial dysfunction attaining values below 50% at 100 *μ*M. Comparing the exposure during 24 or 48 h, mostly 10 *μ*M and 100 *μ*M ROT induced a time-dependent increase in mitochondrial dysfunction (Figure 3(b)).

### 3.4. Tiron and Sodium Salicylate Provided Protection against Paraquat Neurotoxicity

**Figure 4:**
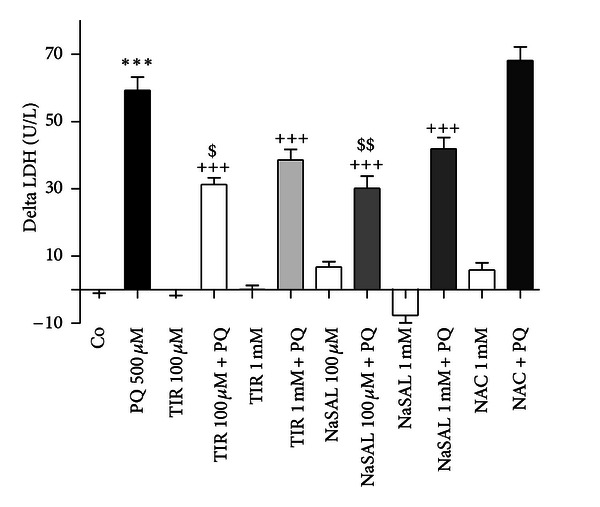
TIR and NaSAL decreased PQ-induced LDH release into the medium, while NAC afforded no protection. Differentiated SH-SY5Y cells were pretreated with TIR (100 *μ*M and 1 mM), NaSAL (100 *μ*M, 1 mM), and NAC (1 mM) 30 minutes prior to cultures exposure to PQ for 72 h. The delta increase in LDH release in units per liter (U/L) into the medium was calculated by subtraction of the respective controls (results were pooled from 3 different experiments, each experiment having 6 different culture wells per condition). The means were compared using one-way ANOVA, followed by Student-Newman-Keuls post hoc test (***P < 0.001 PQ versus control; ^+++^ *P* < 0.001 PQ versus PQ plus protector; ^$^ *P* < 0.05, ^$$^ *P* < 0.01 PQ plus protector low concentration versus PQ plus protector high concentration).

To carry out neuroprotective experiments in human neuroblastoma-differentiated SH-SY5Y cells, we selected the 500 *μ*M PQ concentration and the 72 h exposure period, since under these conditions mitochondrial dysfunction reached about 50% of controls and cell injury was significant. The cells were preincubated with NAC (1 mM), TIR (100 *μ*M and 1 mM), and NaSAL (100 *μ*M, 1 mM, and 10 mM) 30 minutes before exposing cells to PQ. Putative protective drugs alone were also tested for their respective toxicities (Figure 4). 

TIR and NaSAL, both antioxidants, provided partial protection against 500 *μ*M PQ neurotoxicity, as revealed by the LDH assay (Figure 4). The protective action of the referred compounds was only detected in cell injury, revealed by the LDH assay, since these compounds did not offer any protection against mitochondrial dysfunction, as revealed by the MTT assay (data not shown). For both TIR and NaSAL, the protective effect was higher at 100 *μ*M of both protectors comparatively to the 1 mM concentration (Figure 4). Interestingly, NaSAL alone at the 1 mM concentration showed lower LDH release than controls, seemingly to reduce cell death occurring in untreated cells (Figure 4). 

NAC, an antioxidant and glutathione precursor, did not provide any protection against PQ-induced toxicity as revealed both by LDH and MTT assays (Figure 4). 

### 3.5. GBR 12909, Cycloheximide, and L-NAME Provide Protection against Paraquat Neurotoxicity

**Figure 5:**
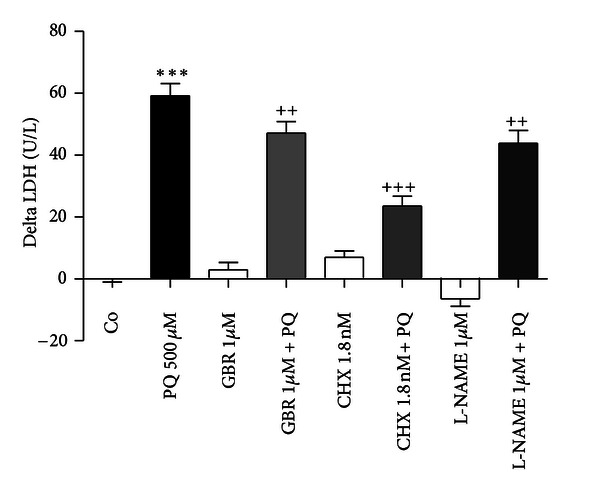
CHX, GBR 12909, and L-NAME decreased PQ-induced LDH release into the medium. Differentiated SH-SY5Y cells were pretreated with GBR 12909 (1 *μ*M), CHX (1,8 nM), and L-NAME (1 *μ*M) 30 minutes prior to cultures exposure to PQ for 72 h. The delta increase in LDH release in units per liter (U/L) into the medium was calculated by subtraction of the respective controls (results were pooled from 3 different experiments, each experiment having 6 different culture wells per condition). The means were compared using one-way ANOVA, followed by Student-Newman-Keuls post hoc test (***P < 0.001 PQ versus control; ^++^ *P* < 0.01, ^+++^ *P* < 0.001 PQ versus PQ plus protector).

GBR 12909 (1 *μ*M), a dopamine transporter blocker, CHX (1.8 nM), a protein synthesis inhibitor, and L-NAME (1 *μ*M), a NOS inhibitor, provided significant protection against PQ neurotoxicity, as revealed by the LDH assay (Figure 5). CHX proved to be the most effective. On the other hand, none of the tested drugs proved protection against mitochondrial dysfunction induced by PQ (data not shown). 

## 4. Discussion

The key findings of our study conducted in differentiated dopaminergic SH-SY5Y cells were as follows: (1) PQ-induced neurotoxicity was concentration and time dependent, promoting a delayed type of cell death with an early mitochondrial dysfunction; (2) MPP^+^ promoted mitochondrial dysfunction, especially at higher times of exposure, but not cellular lyses; (3) ROT-induced neurotoxicity showed a biphasic pattern promoting a delayed type of cell death at later time-points of exposure and higher concentrations; (4) PQ neurotoxicity could be partially prevented by antioxidants, DAT inhibitors, NOS inhibitors, and protein synthesis inhibitors.

MPP^+^, ROT, and PQ are viewed as experimental toxicants to study PD mechanisms *in vitro* [9, 30]. MPP^+^ and ROT are commonly used instead of PQ, because of their well-known mechanisms of toxicity. In addition, studies using MPP^+^ and ROT have already given significant insights into the molecular mechanisms of dopaminergic neuronal death [24, 31]. However, there is a need for more reliable cultured cell models of PD. Several studies using SH-SY5Y cells with these neurotoxicants have been published, without ever having fully elucidated their differential effects, concerning to cell death and optimal concentrations. In the present study, we compared the neurotoxicity of PQ with that of ROT and MPP^+^ using differentiated dopaminergic SH-SY5Y cells. Our data demonstrated that PQ toxicity involved mitochondrial dysfunction and cell death. The increase in LDH release and mitochondrial dysfunction after PQ exposure occurred in a concentration and time dependent manner and was more pronounced at late times of exposure. We verified the existence of two events, an earlier damage to the mitochondrial electronic transport chain that may precede a later membrane burst, typical of cellular lyses. This cellular membrane disruption may be also promoted by external aggressions or other unknown mechanisms promoted by PQ. Our results are in accordance with our analysis, since mitochondrial dysfunction occurred at 24 h and LDH was not measurable at this time point. At higher PQ concentrations and times of exposure, once cells are not intact, the mitochondrial chain is not functional, thus leading to low MTT metabolism.

When compared to MPP^+^, PQ induced a more pronounced mitochondrial dysfunction and cell injury for the same concentrations and time of exposure. Mitochondrial dysfunction may corroborate the hypothesis that PQ, like MPP^+^, inhibits the complex I of the mitochondrial chain [6, 23]. Our study showed that in the same concentration range (100 to 1000 *μ*M) PQ, but not MPP^+^, induced cell lyses and LDH release. This event occurred several hours after mitochondrial dysfunction, since LDH release became only evident after 48 h exposure. In fact, MPP^+^ exposed cells might have lost their metabolic capacity but yet not their membrane integrity, suggesting delayed cell death. Not only SH-SY5Y-differentiated cells seem to be more susceptible to PQ but also PQ is more stable in solution than MPP^+^, which is photosensitive and may lose activity when in solution with longer exposure periods. Therefore, PQ may represent several advantages over MPP^+^ to study Parkinson's mechanisms *in vitro*, namely, when using cultured cells.

Concerning ROT, a widely used toxicant to promote dopaminergic toxicity, it showed a biphasic pattern of neurotoxicity. Our results are not in agreement with those presented by Molina-Jimenez [19], who observed that ROT caused a proportional time- and dose-dependent decrease in SH-SY5Y cellular viability. This can be explained by a different cell culture protocol, namely, their FBS removal and lower cellular density. Herein, ROT, at 1 *μ*M for 48 h, reduced cell lyses and promoted lower LDH leakage compared to control cells, an effect no longer seen at latter times of exposure. On the other hand, the MTT assay proved mitochondrial dysfunction after 48 h exposure to 1 *μ*M. This may be connected to a late death pattern, as ROT is postulated to be a high affinity complex I inhibitor [7] and to induce apoptosis via activation of caspase 3 apoptosis pathway [20]. In fact, ROT binds near to the quinone-binding site and blocks electron transport, preventing NADH from being converted to ATP; in this way the proton pumping is compromised, which culminates in increased superoxide generation, and consequent cell death [32]. Cell membrane rupture occurs several hours after compromising the metabolic activity at lower concentrations, though at higher concentrations it may demonstrate toxicity by other means [8]. Other studies showed that ROT causes apoptosis at low doses and necrosis when applied at high doses, thus corroborating our results [33]. We demonstrated in SH-SY5Y cells that PQ promoted a more defined pattern of time-concentration-dependent cell death; meanwhile, ROT induced a biphasic pattern of toxicity. Moreover, PQ chloride salt is water soluble, while ROT has to be prepared in DMSO, which is an hazardous substance. Therefore, PQ may represent advantages over ROT to study Parkinson's mechanisms *in vitro*, at least in SH-SY5Y-differentiated cells.

Comparing the toxicity of ROT and MPP^+^ in SH-SY5Y-differentiated cells, one can conclude that ROT promoted a higher grade of mitochondrial dysfunction and cell injury than MPP^+^. In fact, 10 times lower concentrations of ROT and shorter exposure times were required for achieving the degree of toxicity seen with MPP^+^. One possible explanation may be that ROT does not need a transporter to enter the cells as it crosses membranes by diffusion [7]. Our results are in accordance with Richardson and collaborators who demonstrated that higher MPP^+^concentrations were required to achieve the same degree of complex I inhibition, showing that ROT has a higher complex I affinity [8]. In contrast to ROT, the concentrations of MPP^+^ used in our SH-SY5Y-differentiated cells did not result in cell lyses during the 72 h of exposure. Other studies using SH-SY5Y have reported cell death using higher concentrations of MPP^+^, up to 10 mM [22, 34, 35]. Moreover, differentiated SH-SY5Y cells have an enhanced resistance towards neurotoxins. Our group recently showed that differentiation of SH-SY5Y cells with RA and TPA led to SH-SY5Y neurons with higher ability to accumulate dopamine and higher resistance towards dopamine neurotoxicity [18]. Importantly, a study by Cheung and coauthors demonstrated that differentiation of SH-SY5Y cells with RA conferred higher resistance towards MPP^+^ toxicity [36]. Thus, using higher concentrations of MPP^+^and longer periods of exposure should be necessary to obtain significant cell lyses.

Our study demonstrated that several compounds could partially prevent or slow PQ-induced toxicity. NaSAL is known to prevent PQ-induced apoptosis in the rat lung [37]. In fact, one of the proposed mechanisms of PQ neurotoxicity is oxidative stress by producing ROS such as superoxide anion (O_2_^•−^), hydroxyl radicals (OH^•^), and hydrogen peroxide (H_2_O_2_), which increase lipid peroxidation, increase oxidation of proteins, DNA and iron content and promote a marked decrease in reduced glutathione (GSH) and GSH/glutathione disulfide ratio [23, 38]. Oxidative stress is also a typical feature of PD brains [39]. Salicylates are important HO^•^ scavengers and therefore reduce PQ toxicity [37]. On the other hand, salicylates may also form stable complexes with PQ [40]. These two proposed mechanisms may be the explanation of the neuroprotection obtained by us with NaSAL against PQ-induced injury. Interestingly, NaSAL did not prevent mitochondrial dysfunction. On the other hand, LDH values were reduced, possibly because direct cellular membrane damage was prevented, either by complex forming or oxidative radical reduction. Still PQ may promote toxicity by other means, once neurotoxicity was not fully prevented. 

Another antioxidant used, TIR, partially prevented PQ-induced neurotoxicity. TIR is a known scavenger of O_2_^•−^, acting both at extracellular and intracellular levels. O_2_^•−^ that originated from PQ redox cycle can be scavenged by TIR, therefore affording protection. Also, O_2_^•−^ can be converted further to H_2_O_2_ and to HO^•^ through Fenton-type reactions, using Fe^2+^, promoting more oxidative stress [41]. However, TIR only partially prevented cell lyses and not mitochondrial dysfunction promoted by PQ, which may indicate that its neurotoxicity occurred by additional mechanisms.

PQ-induced cell death might occur through the formation of reactive nitrogen species (RNS), which also promote oxidative stress. The NOS inhibitor L-NAME also attenuated PQ toxicity. Nitric oxide produced by NOS forms peroxynitrite anion, by reacting with O_2_^•−^ produced in the redox-cycle of PQ, crosses membranes, and can easily oxidize proteins, lipids, RNA, and DNA [42]. Overall the protective effects of the two antioxidants and the NO synthesis inhibitor corroborate the hypothesis that oxidative stress is a major mechanism of PQ neurotoxicity [23, 42, 43].

Our study revealed that CHX, a protein synthesis inhibitor, protected against PQ-induced neurotoxicity. Therefore, PQ-induced cell death might involve proteins, whose synthesis is prevented by CHX. Protein synthesis is involved in many processes of programmed and nonprogrammed cell death, and therefore PQ-induced dopaminergic death might involve those processes [44].

GBR 12909 conferred partial protection against PQ-induced neurotoxicity revealing that PQ entrance into the dopaminergic neuron might occur through the DAT transporter, which is corroborated by Yang and Tiffany-Castiglioni [23]. Since PQ is structurally very similar to MPP^+^, it may as well use dopamine transporters [45], though this still remains a controversial issue, as there are contrary opinions like that of Richardson and collaborators [8], who claim that PQ toxicity is independent of DAT expression or Ramachandiran and coauthors [46], who claim that PQ does not require a functional dopamine transporter for dopaminergic toxicity. In fact, we found that despite DAT blocking, PQ still exerts neurotoxicity, so it is suggested that it may nevertheless enter the cell or exert toxic effects on the outside leading to cellular lyses.

Other studies have evaluated the effects of the pharmacological agents that we used against PQ neurotoxicity to circumvent MPP^+^ or ROT neurotoxicity. L-NAME did not present any significant protection to MPP^+^ toxicity in SH-SY5Y cells [47], whereas GBR 12909 blocked its neurotoxic effects [17]. Meanwhile, CHX reduced proapoptotic proteins production in response to MPP^+^-induced toxicity [48], and also CHX blocked MPP^+^-induced cell death in the dopaminergic cell line MN9D [49]. In rats, NaSAL significantly attenuated striatal dopamine depletion caused by intrastriatal MPP^+^ infusion [50]. In what concerns ROT, CHX was demonstrated to reduce cell death in cultured cortical neurons [51]. In another study, GBR 12909 significantly prevented the ROT-induced decrease of cell viability in PC12 cells [52]. In rats, NaSAL demonstrated neuroprotective efficacy against ROT toxicity [53]. Also, in oral cancer cell lines ROT-induced apoptosis was inhibited by TIR [54]. 

## 5. Conclusions

The results obtained in the present study showed that PQ promoted a diverse profile of toxicity when compared to MPP^+^ and ROT and could present advantages over those two toxicants for modelling PD *in vitro*. In fact, PQ in differentiated dopaminergic SH-SY5Y cells promoted a concentration- and time-dependent toxicity, promoting a delayed type of cell death with mitochondrial dysfunction leading to cellular lyses. We further studied the mechanisms of PQ-induced toxicity and demonstrated that antioxidants, NOS inhibitors, DAT inhibitors, and protein synthesis inhibitors could be useful tools in preventing dopaminergic toxicity, at least *in vitro*. 

## Abbreviations

CHX: Cycloheximide
DAT: Dopamine transporter
D-MEM: Dulbecco's Modified Eagle Media
DMSO: Dimethyl sulfoxide
GBR 12909: 1-(2-[Bis(4-fluorophenyl)methoxy]ethyl)-4-(3-phenyl-propyl)piperazine
GSH: Reduced glutathione
H_2_O_2_: Hydrogen peroxide
LDH: Lactate dehydrogenase
L-NAME: *N *_*ω*_-Nitro-L-arginine methyl ester hydrochloride
MPP^+^: 1-Methyl-4-phenyl-pyridinium ion
MPPP: 1-Methyl-4-phenyl-4-propionoxy-piperidine
MPTP: 1-Methyl-4-phenyl-1,2,3,6-tetrahydropyridine
MTT: 3-[4,5-Dimethylthiazol]-2,5-diphenyltetrazolium
NAC: *N*-Acetylcysteine
NADH: Nicotinamide adenine dinucleotide reduced form
NaSAL: 2-Hydroxybenzoic acid sodium salt
NEAA: Nonessential amino acids
NOS: Nitric oxide synthase
O_2_^•−^: Superoxide anion
OH^•^: Hydroxyl radical
PBS: Phosphate-buffered saline
PD: Parkinson's disease
PQ: Paraquat
RA: Retinoic acid
RNS: Reactive nitrogen species
ROS: Reactive oxygen species
ROT: Rotenone
SDS: Sodium dodecyl sulfate
SNpc: Substantia nigra pars compacta
TIR: Tiron
TPA: 12-O-Tetradecanoyl-phorbol-13-acetate.
